# Structural and compositional analysis of a casting mold sherd from ancient China

**DOI:** 10.1371/journal.pone.0174057

**Published:** 2017-03-15

**Authors:** Yunbing Zong, Shengkun Yao, Jianfeng Lang, Xuexiang Chen, Jiadong Fan, Zhibin Sun, Xiulan Duan, Nannan Li, Hui Fang, Guangzhao Zhou, Tiqiao Xiao, Aiguo Li, Huaidong Jiang

**Affiliations:** 1 State Key Laboratory of Crystal Materials, Shandong University, Jinan, Shandong, China; 2 School of Physical Science and Technology, ShanghaiTech University, Shanghai, China; 3 Department of Archaeology, Shandong University, Jinan, Shandong, China; 4 S hanghai Synchrotron Radiation Facility, Shanghai Institute of Applied Physics, Chinese Academy of Sciences, Shanghai, China; Northwestern University Feinberg School of Medicine, UNITED STATES

## Abstract

Casting had symbolic significance and was strictly controlled in the Shang dynasty of ancient China. Vessel casting was mainly distributed around the Shang capital, Yin Ruins, which indicates a rigorous centralization of authority. Thus, for a casting mold to be excavated far from the capital region is rare. In addition to some bronze vessel molds excavated at the Buyao Village site, another key discovery of a bronze vessel mold occurred at Daxinzhuang. The Daxinzhuang site was a core area in the east of Shang state and is an important site to study the eastward expansion of the Shang. Here, combining synchrotron X-rays and other physicochemical analysis methods, nondestructive three-dimensional structure imaging and different elemental analyses were conducted on this mold sherd. Through high penetration X-ray tomography, we obtained insights on the internal structure and discovered some pores. We infer that the generation of pores inside the casting mold sherd was used to enhance air permeability during casting. Furthermore, we suppose that the decorative patterns on the surface were carved and not pasted onto it. Considering the previous compositional studies of bronze vessels, the copper and iron elements were analyzed by different methods. Unexpectedly, a larger amount of iron than of copper was detected on the surface. According to the data analysis and archaeological context, the source of iron on the casting mold sherd could be attributed to local soil contamination. A refined compositional analysis confirms that this casting mold was fabricated locally and used for bronze casting.

## Introduction

The origin of the Bronze Age in China may date back to the Majiayao culture [1,2], whereas the fabrication and use of bronze vessels prevailed in the Shang dynasty [3]. In northern China, many bronze vessels have been discovered around Yin Ruins, the capital of the later Shang dynasty [4,5]. Bronze vessel production seems to have been concentrated in the capital region and that these reported in the paper are the first vessel casting mold remains discovered outside of Anyang for the Anyang period [6,7]. In 2007, several bronze molds and vessels were excavated at the Buyao Village site [8,9]. Bronze vessels were highly significant markers of status and crucial media for ritual communication with the ancestral powers, and so the discovery of mold fragments outside of the Great Settlement Shang is highly significant.

**Fig 1:**
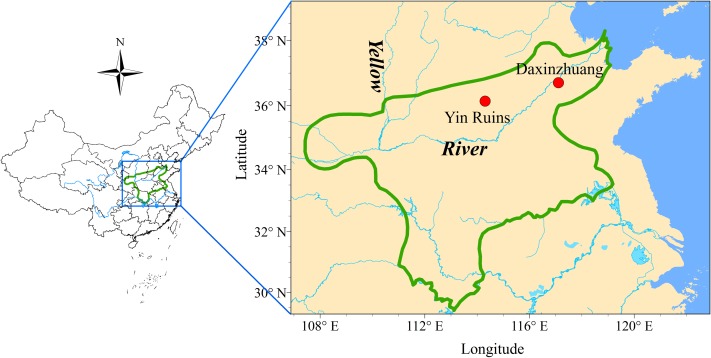
Map of the Shang Dynasty and location of Daxinzhuang. Both Yin Ruins and Daxinzhuang sites are marked in the map. Reprinted from [11] under a CC BY license, with permission from [Lamassu Design], original copyright [2009].

Daxinzhuang is east of Yin Ruins and existed in the middle or late Shang dynasty (Fig 1) [10–13]. It is located downstream of the Yellow River and is famous for the excavation of the Oracle Bone inscriptions of the Shang dynasty that have been found outside of Yin Ruins [14]. Daxinzhuang is a typical central territorial settlement and is one of the largest sites in the Haidai area [15,16]. The Daxinzhuang site is considered an important core area in the eastern region of Shang state. Moreover, these casting mold sherds may give clues of eastward expansion in the Shang regime [17,18].

A key point in the use of casting molds is that bronze vessels could not be produced without molds in ancient China [4,19,20]. The manufacturing process of casting molds has been previously discussed [21–23]. As the process of casting is using a mold which contains a hollow cavity of the desired shape, in which the liquid metal is poured into to get a bronze ware after solidification. By referring to the decoration on the surface of the casting molds, archaeologists usually adopt the morphology method [5,19,20]. To determine the decorative structure and manufacturing process of the mold, X-ray computed tomography (XCT), a widely used nondestructive method in archaeology [24–27], is used to investigate a casting mold sherd. X-ray fluorescence mapping (XFM) is a two-dimensional elemental characterization method that obtains elemental mapping on the surface of materials [21,28–31] and is used to confirm a casting mold’s origin and whether it has been used. Inductively coupled plasma atomic emission spectrometry (ICP-AES) traces elements at a higher precision [32–34]. X-ray photoelectron spectroscopy (XPS) and energy dispersive spectrometer (EDS) are other methods that are generally used for compositional analysis [35–38]. In this work, different methods compensated for one another to complete a final structural and compositional analysis.

## Materials and methods

**Fig 2:**
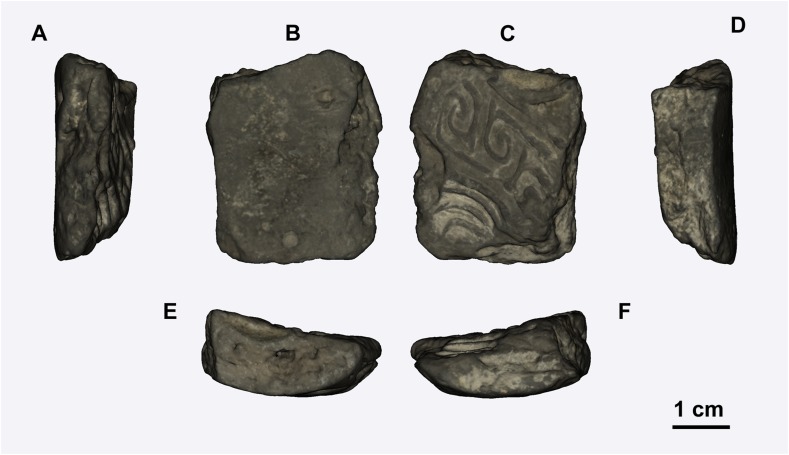
Different views of the casting mold sherd. (A-F) Six faces of the casting mold sherd.

The casting mold sherd in this study comes from the Daxinzhuang site, which is located in the eastern part of Shang state. Although large amounts of bronze wares have been excavated here, few casting molds have been found. Importantly, a piece of casting mold with fine decoration was obtained. The casting mold sherd (sample number: 2010DXZJ9T2024H900②:18) was excavated in pit H900 at Daxinzhuang site and other remains including pottery sherds and animal bones were also excavated (S1 File). We infer that it is a vessel casting mold sherd from the arc-shaped front and back (Fig 2E and 2F), because the molds used to create bronzes with a shape of cambered surface. To confirm the age of the casting mold sherd, a pig bone found in the same excavation unit was used for standard AMS-^14^C dating in a Beta Analytic laboratory (S1 Fig). We also collected and studied the excavated pottery pieces that were made in local styles of the Daxinzhuang site to confirm the elements that are present in the casting mold sherd (S2 Fig).

Synchrotron X-ray tomographic microscopy was used to study the three-dimensional structure of the casting mold sherd, particularly to understand the relation between the decoration and the casting mold sherd matrix. Because of its short wavelength, X-rays have strong penetration ability and allow high-resolution imaging. Compared with laboratory X-ray sources, synchrotron X-rays have a higher flux, brightness and signal-to-noise ratio.

To confirm the composition of the casting mold sherd, XPS, ICP-AES, EDS and XFM were conducted. Before these tests, the outermost layer of the entire mold was cleaned using a soft hairbrush with ethyl alcohol to eliminate external contamination. The sampling position was located in the back of the casting mold sherd to avoid the possible influence of the casting elements that are present in the decorative side. To further avoid some other surface contamination, the near-surface region whose depth was about 1mm was sampled. Then a knife was used to scrape the powder from the casting mold sherd. 0.2g, 0.1g and 0.01g of powder were respectively prepared for XPS, EDS and ICP-AES experiment. XFM was performed to directly detect the casting-related elemental information on the decorative side.

## Results and discussion

### AMS-^14^C dating

Standard AMS-^14^C dating was performed on a pig bone from the same context as the sherd to confirm the age of the casting mold sherd. The obtained results are presented in Table 1. The age of the collected pig bone is more than 3,000 years old and suggests that the age of the casting mold sherd obtained in Daxinzhuang is approximately 3,000 years old, which dates to the late Shang and the early Western Zhou.

**Table 1:** AMS-^14^C dating of archaeological pig bone.

Lab number	Location	Conventional ^14^C age B.P.	2 Sigma Calibrated age B.P. (95% Probability)
Beta—418905	Daxinzhuang	2,960 ± 30	3,210–3,005

The half-life of ^14^C is 5,568 years and B.P. refers to the date before 1,950.

### Three-dimensional structure of casting mold sherd and technology analysis

**Fig 3:**
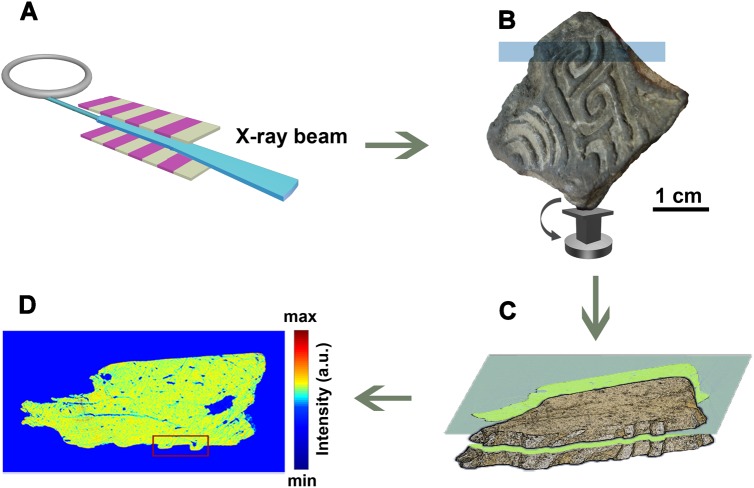
Schematic of nondestructive 3D X-ray tomography. (A) X-ray beam generated by synchrotron source. (B) The casting mold sherd was positioned on a rotation sample stage. The blue shaded region indicates a portion exposed to the X-ray beam. (C) A slice was extracted from the reconstructed result of the X-ray exposed region. (D) The extracted slice was displayed in red-green-blue mode, and raised decorated patterns in the casting mold sherd were marked by a red rectangle. The color bar indicates the level of intensity.

To investigate the structure of the decoration and casting technology, synchrotron XCT was performed at the Shanghai synchrotron radiation facility (Fig 3). Considering the size of the casting mold sherd and the experimental field of view, one representative section, indicated by the blue-shaded region, was selected. The three-dimensional reconstructed result reveals not only the surface information but also the core structure of the sample. According to the principle of absorption X-ray imaging, the reconstructed intensity represents the mass or density of the constituents that are present in the casting mold sherd [39]. Fig 3D shows one slice that was extracted from the imagery of the casting mold sherd. Isolated large-sized pores and crack traces can be clearly recognized in Fig 3D. The small pores that are distributed in different parts of the casting mold sherd suggest that these structures were not formed randomly, but were deliberately produced due to the need for good air permeability during casting [20,23]. This premise suggests that the mold manufacturing technology was well developed.

**Fig 4:**
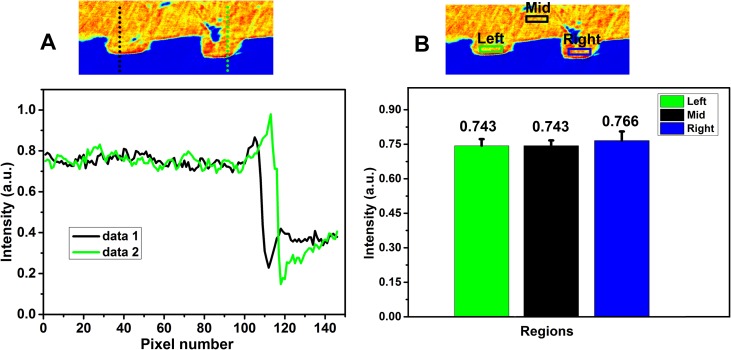
Structural analysis of decorated strips in casting mold sherd. (A) Line scan on the red rectangle region from Fig 1D. The dotted lines in the raised strips and corresponding intensity contour the plots below. (B). Bar diagram of the average intensity obtained from the regions in two raised strips and the space inside the casting mold sherd. Three regions with the same size were selected from two raised decorated patterns (marked by green and blue rectangles) and one deep inside the casting mold sherd (marked by black rectangle).

Because the structure connecting the raised decoration and the casting mold sherd matrix has attracted much attention in archaeological metallurgy, a zoom-in analysis was performed in the region that is marked by a red rectangle in Fig 3D [23,40]. A line scan was used to differentiate the body of the casting mold sherd and the decorated patterns (Fig 4A).

The plot of intensity in two positions is shown as data1 (black line) and data2 (green line) in Fig 4A. Near pixel number 110, a steep jump in the two lines corresponds to the boundary between the air and the casting mold sherd. The lines are flat in the range from pixel numbers 0 to 100, which suggests that there is no boundary between the casting mold sherd body and the decorated patterns. This result reveals that the decorated patterns were not pasted but carved on the pottery to make the mold. The compositional difference among the decorated patterns on the casting mold sherd and pottery body was determined by scanning the three different regions (left, middle and right) of the casting mold sherd and calculating the average region intensity (Fig 4B). These equirotal regions (13×60 pixels) were selected from two raised decorated patterns (marked by green and blue rectangles) and the inside of the casting mold sherd (marked by a black rectangle). The pixel size is 0.2 mm. The average intensity value of the two decorated patterns is 0.743 and 0.766 (arbitrary unit), respectively. The corresponding intensity value inside the casting mold sherd is 0.743. The minimum intensity difference between the decorated patterns and the casting mold sherd body confirms that the decorated patterns were designed by being carved out of the casting mold.

### Compositional analysis of the casting mold sherd

#### XPS analysis

**Fig 5:**
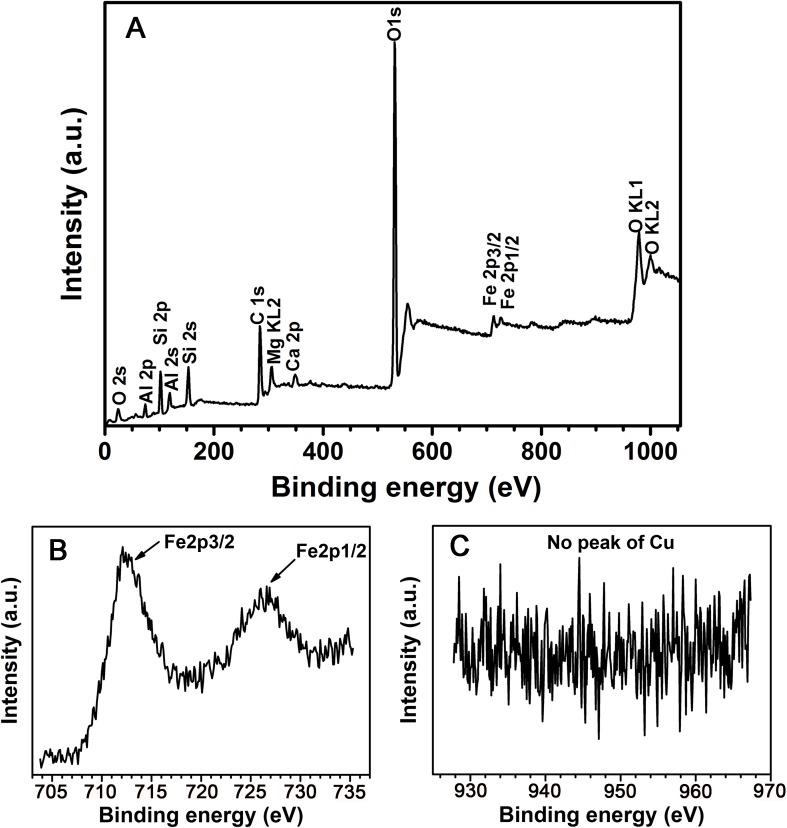
X-ray photoelectron spectroscopy of near-surface powder. (A) The survey XPS spectra show the general elementary composition. (B) High resolution scan of the iron element. The double peak of Fe 2p orbit can be recognized. (C) High resolution scan of the copper element with no corresponding peaks. A comparison between (B) and (C) proves that the iron content in the casting mold sherd is much greater than copper.

The powder used for the XPS experiment was sampled from the back of the casting mold sherd and the sampling depth was about 1mm to avoid surface contamination. 0.2 g of powder sample was collected for the survey of XPS spectra and high-resolution photoelectron spectra. We determined the survey XPS spectra of the powder from the casting mold sherd (Fig 5A). Oxygen, silicon, calcium, carbon, aluminum, magnesium and iron elements were detected in the casting mold sherd. The C1s peak at 284.6 eV was due to contaminated carbon. To identify the content of copper and iron in the sample, the high-resolution photoelectron spectra of Cu and Fe were measured (Fig 5B and 5C). The presence of iron is identified at two peaks, namely, 712.1eV and 726.6 eV, which correspond to Fe2p_3/2_ and Fe2p_1/2_ respectively; whereas no peaks were observed for copper. The XPS result of the pottery made in local styles is presented in S3 Fig. No significant compositional differences were found between these mold pieces and the casting mold sherd, which indicates that the casting mold was probably made locally.

#### EDS analysis

The sampling procedure in EDS experiment was similar to that in the XPS experiment. 0.1g of sample powder was prepared. The EDS result (Table 2) shows that the elements presented in the sample are mainly carbon, oxygen, magnesium, aluminum, silicon, potassium and iron. However, calcium that was found by XPS was not observed in EDS result. A small amount of potassium that was not detected by XPS was observed in EDS result. This is because that the difference of sensitivity between XPS and EDS as well as the nonuniformity of the casting mold sherd may lead to the inconformity. Importantly, the EDS result further confirms the presence of iron in the casting mold sherd.

**Table 2:** EDS results of the elemental analysis of the casting mold sherd.

Elements	Weight (%)	Atomic (%)
C	4.18	6.46
O	61.07	70.80
Mg	2.18	1.66
Al	10.59	7.28
Si	19.02	12.56
K	1.75	0.83
Fe	1.21	0.40
Total	100	100

#### ICP-AES analysis

ICP-AES determines the composition of elements with greater accuracy. A powder of the casting mold sherd 0.01 g, was dispersed in 10 ml of HNO_3_ solution. The elements that were traced from the ICP-AES measurement are presented in Table 3. In contrast to the EDS and XPS results, ICP-AES detected a small amount of copper in the casting mold sherd. Furthermore, the presence of iron in the casting mold sherd was 24.1 times greater than that of copper, whereas the corresponding value from the pottery made in local styles was approximately 145.6. This finding indicates that the copper content in the casting mold sherd is greater than that in other pottery pieces. The tiny difference proves that although it was made locally, some special fabrication procedure changed the composition of the casting mold. Additionally, a possibility that the higher copper content in the casting mold sherd may be from the casting residues on the mold sherd should not be excluded completely. The iron that is present in the casting mold sherd may be from the contamination of the native soil.

**Table 3:** ICP-AES results of the elemental analysis of the casting mold sherd (indicated as CMS) and the archaeological pottery excavated from Daxinzhuang (indicated as APD).

Elements	Content (ppm)	
	CMS	APD
Mg	0.547	2.472
Al	0.756	1.114
Si	0.414	2.160
Ca	3.382	15.170
Fe	1.328	39.040
Cu	0.055	0.268

#### X-ray fluorescence mapping of copper and iron

**Fig 6:**
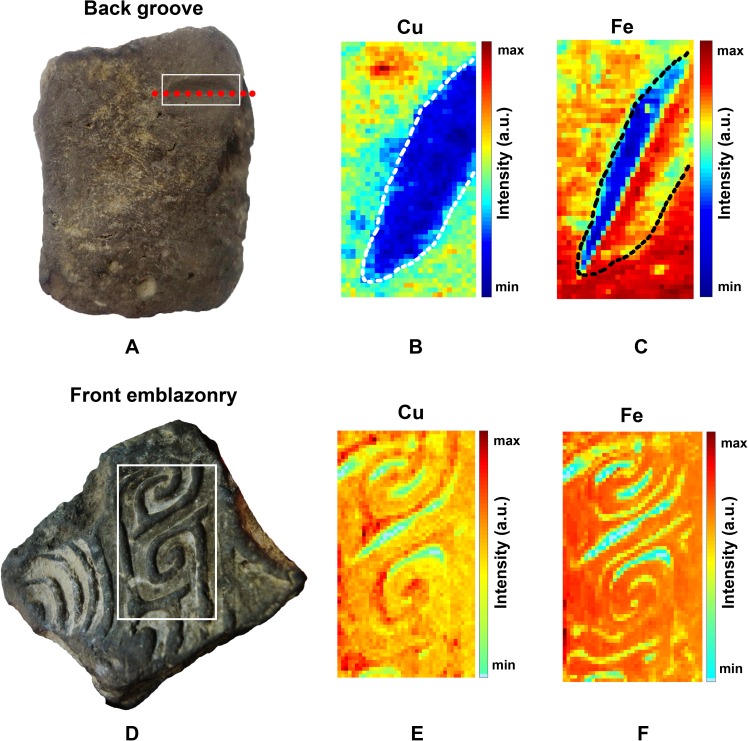
X-ray fluorescence mapping of copper and iron mapping in front decoration and back groove. (A) A groove is labelled by a white rectangle on the back of the casting mold sherd. One red dashed line indicates the deepest position in the groove. (B) X-ray fluorescence mapping of the copper on the back and a white dashed line encircles the groove. (C) X-ray fluorescence mapping of the iron on the back and a black dashed line encircles the groove. One side of the deepest line shows a gathering of iron, and on the other side, this reverses. (D) A probing range is labelled by a white rectangle on the front of the casting mold sherd. (E), (F) X-ray fluorescence mapping on the front decoration of copper and iron, respectively. Both elements have an uneven distribution and some correspondence to the profile of decorated patterns.

Elemental mapping on the pattern side of the casting mold sherd is helpful to understand the casting technology. Focusing on copper and iron, X-ray fluorescence was conducted to finish the two-dimensional elemental mapping for both sides of the casting mold sherd (Fig 6). The color bar indicates the fluorescence signal intensity that is emitted from copper and iron that is collected by the detector. The XPS, EDS and ICP-AES analyses studied powders from the near-surface, but a position deeper inside the mold completely reveals the core components of the casting mold sherd. To reveal the inside of the casting mold sherd, a long shallow groove was dug in the back and labeled by a white rectangle (Fig 6A). The distributions of copper and iron are shown in Fig 6B and 6C, respectively. The copper content within the region (dashed line) is reduced compared with the copper content in other positions. The distribution of iron in Fig 6C is different from the distribution of copper in Fig 6B. In the groove, the deepest red dashed line separates the distribution of iron into two regions (Fig 6A). One side of the line shows an enrichment of iron, whereas the reverse is found on the other side. This result can be explained by the process of digging. Iron in one face of the groove was pushed into the other face, which leads to enrichment. Overall, this finding indicates that there is little copper and iron that constitutes the pottery matrix. However, copper and iron exist on the back, and more iron may exist on the surface of the casting mold sherd. In addition, copper and iron were probed on the front decoration side (Fig 6D), and their distributions are shown in Fig 6E and 6F, respectively. The mapping results show that the front has a more homogenous element distribution. In some positions, the elemental trace may be related to the profile of the decorated patterns, and it may be caused by casting.

**Fig 7:**
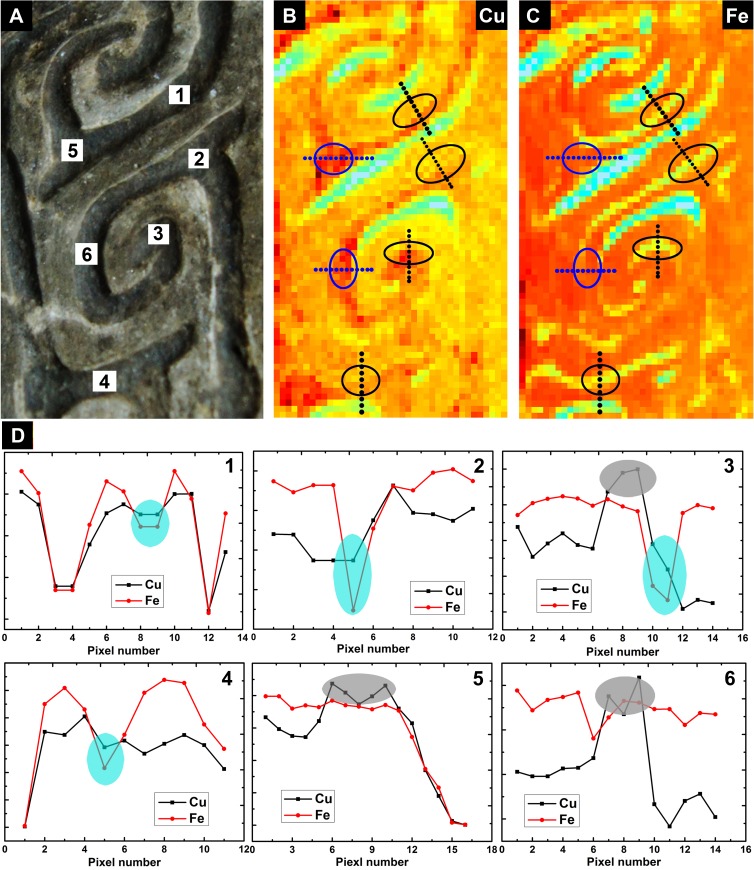
Line scan contour plot of XRF mapping of copper and iron. Six selected regions in the decorative side (A) and corresponding line scan image for Copper (B) and Iron (C). The corresponding positions in (B) and (C) are tagged with grey ellipses (positions 1, 2, 3, and 4) and blue ellipses (positions 5 and 6). Dotted straight lines across the ellipses indicate the line scan directions. (D) The line scan results are displayed in plots 1–6. Normalization was performed on both copper and iron fluorescence intensity before the line scan. Four sky-blue ellipses were drawn on the line scan results (1, 2, 3, and 4), and three ash ellipses were drawn on the line scan results (3, 5, and 6). The sky-blue ellipses reveal a steep reduction of iron, and the grey ellipses show an enrichment of copper.

To conduct further accurate analyses, line scan was performed and is shown in Fig 6E and 6F, and the results are presented in Fig 7. According to the distribution of copper and iron in Fig 6, six representative positions were selected from the decorative patterns (Fig 7A). Then, the corresponding positions in copper and iron fluorescence mapping were determined. According to the fluorescence intensity distribution, positions 1, 2, 3, and 4 were encircled by grey ellipses, and positions 5 and 6 were encircled by blue ellipses (Fig 7B and 7C). A line scan was subsequently performed on the selected regions. The dotted straight lines across the ellipses indicate the direction of the line scan. The fluorescence mapping intensity was normalized to the range of 0~1 to compare copper and iron. The line scan results of different positions on the decoration side are shown in Fig 7D 1–6, which correspond to positions 1–6, respectively. The blue shaded portions in the line scan results of positions 1, 2, 3 and 4 suggest that there are four valleys in the raised decorated patterns, which indicates a reduction of iron. As discussed in the ICP-AES analysis, the iron that is present on the casting mold sherd may be from the native soil, and the iron reduction on the raised decorated patterns may be explained by incidental scrubbing and cleaning work during excavation. However, this explanation is not the case for copper because elemental enrichment of copper was discovered on the raised decorated stripes. The peaks that are indicated by the gray-shaded portions in the line scan results (Fig 7D 3, 5, and 6) also show the enrichment of copper. Therefore, this mold was likely used for bronze casting, but not for iron casting.

## Conclusions

In this work, we investigated one casting mold sherd that was excavated from Daxinzhuang. The standard AMS-^14^C dating confirms that it is a mold from the Shang dynasty. The nondestructive X-ray structural and technological analysis shows that this casting mold was carefully designed for casting. The pores that were discovered inside indicate that the people at the time were aware of the air permeability issue for casting molds. The three-dimensional structure analysis also shows that the decorated patterns on the surface were carved into the mold. Comprehensive elemental analysis methods combined with other excavations in Daxinzhuang prove that this mold was made locally and used for bronze vessel casting. By referring to previous excavations of objects connected with the royal capital, such as bronze articles, jadeware and oracular bones, the casting mold sherd could verify that the Daxinzhuang site was an important regional center in the late Shang state. This clue helps archaeologists to understand the cultural prosperity that was downstream of the Yellow River 3,000 years ago. However, more excavations will be helpful and necessary to reveal more details about the expansion of the late Shang state.

## Supporting information

S1 Fig Preserved archaeological pig bone extracted from the same excavation unit. (TIF)

S2 Fig Excavated pottery pieces made in local styles. Large amounts of pottery were excavated at the Daxinzhuang site. This pottery represents the texture of the native soil at the Daxinzhuang site.
(TIF)

S3 Fig XPS spectrum of pottery pieces excavated at the Daxinzhuang site (TIF)

S1 File Permission information of Fig 1 and the Daxinzhuang site. (PDF)
