# Supplementary figures and images for: Structural and compositional analysis of a casting mold sherd from ancient China
Source: PLoS One. 2017 Mar 15;12(3):e0174057. doi: 10.1371/journal.pone.0174057 (PMC5352019; doi:10.1371/journal.pone.0174057)

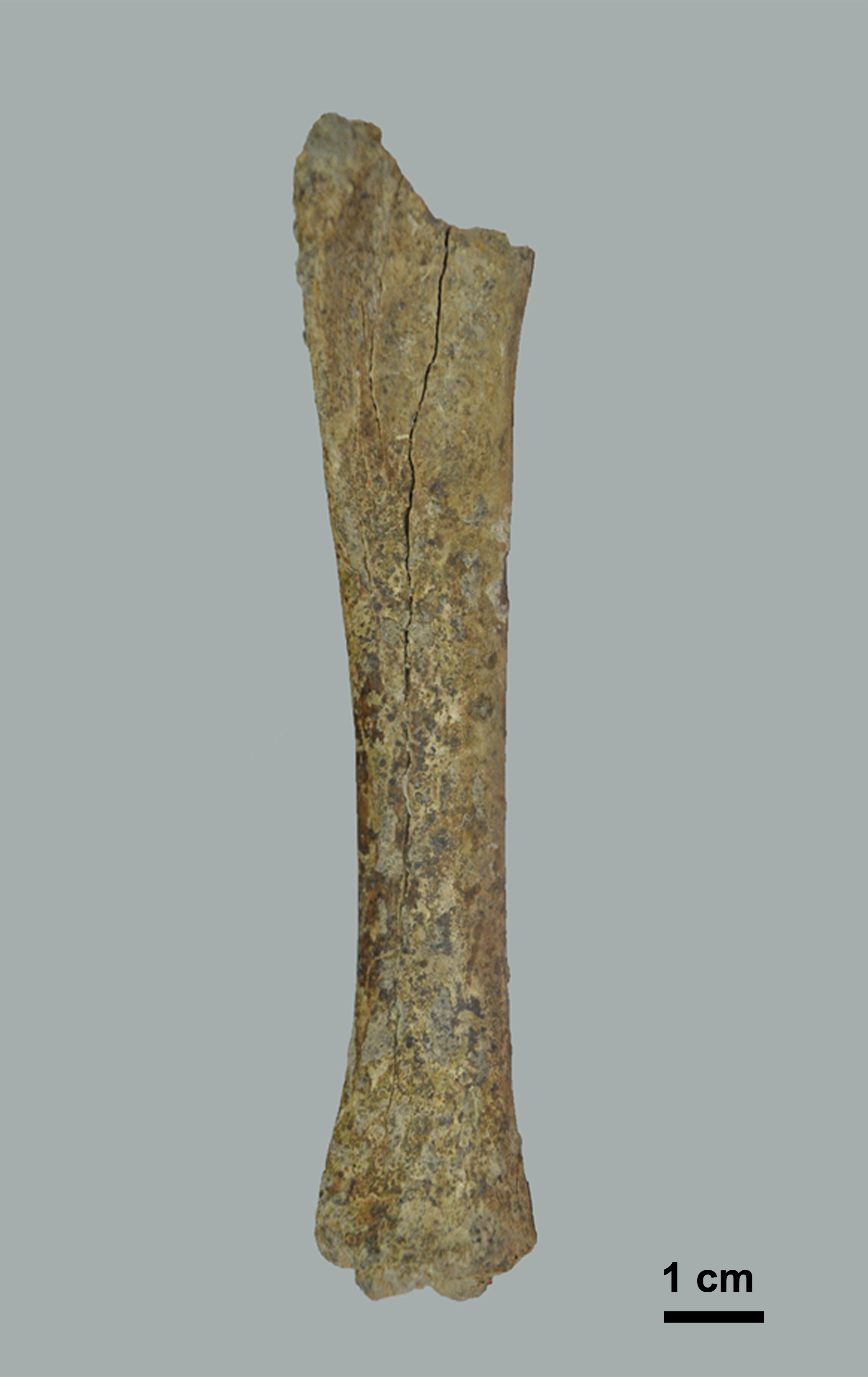

Supplement: S1 Fig — (TIF) [file pone.0174057.s001.tif]

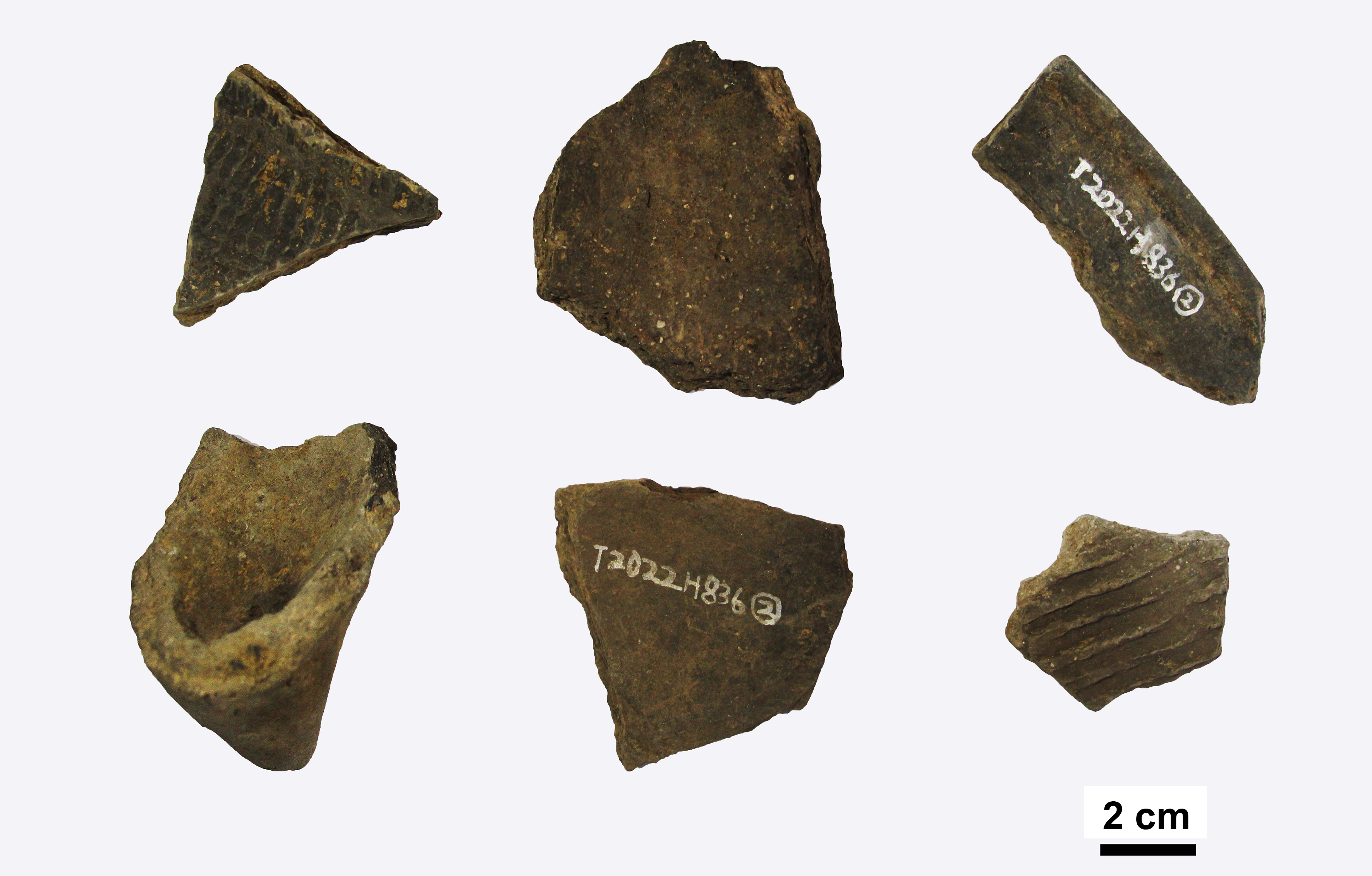

Supplement: S2 Fig — Large amounts of pottery were excavated at the Daxinzhuang site. This pottery represents the texture of the native soil at the Daxinzhuang site. (TIF) [file pone.0174057.s002.tif]

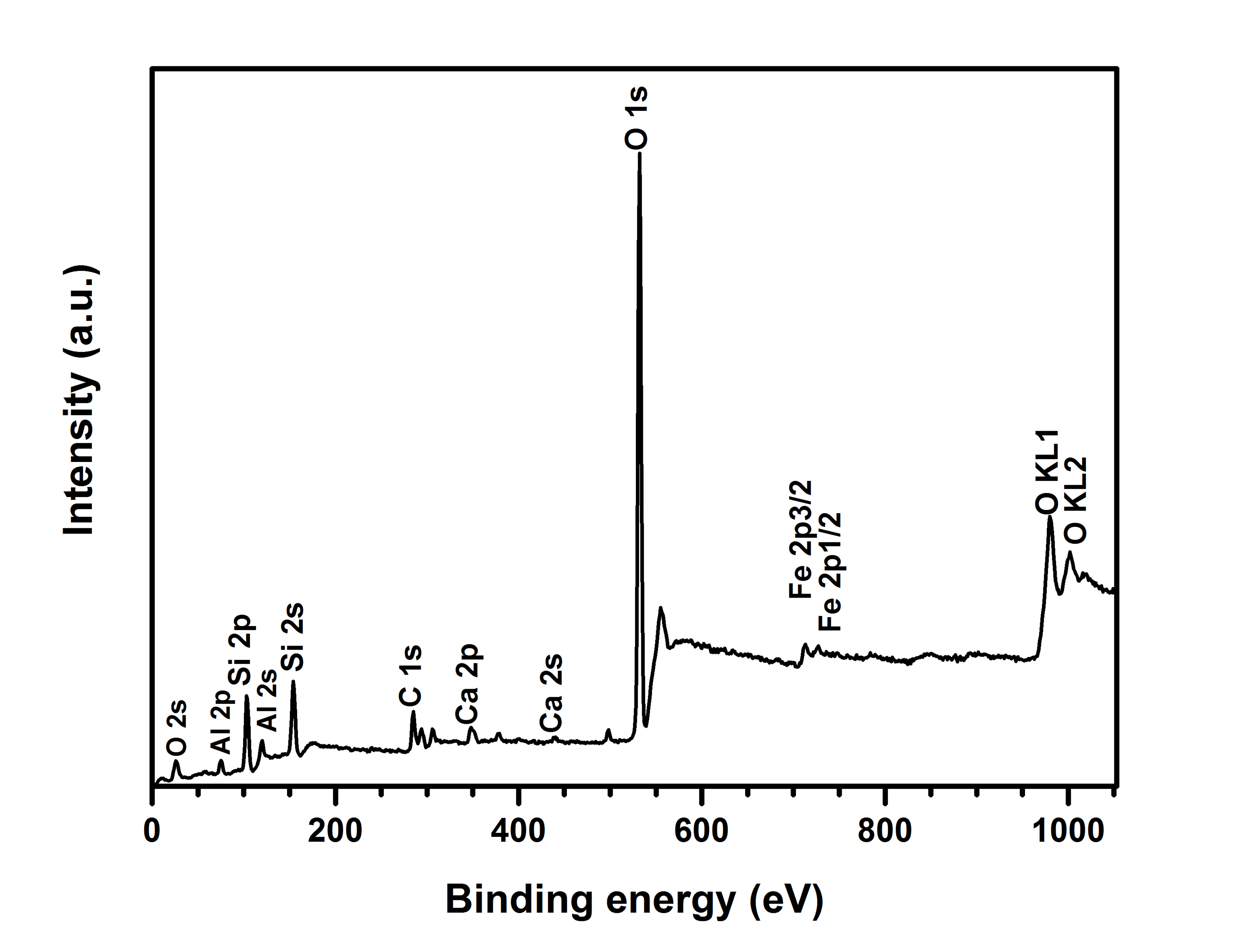

Supplement: S3 Fig — (TIF) [file pone.0174057.s003.tif]
