# Supplementary material for: Structural and compositional analysis of a casting mold sherd from ancient China
Source: PLoS One. 2017 Mar 15;12(3):e0174057. doi: 10.1371/journal.pone.0174057 (PMC5352019; doi:10.1371/journal.pone.0174057)
Supplement: S1 File — (PDF) [file pone.0174057.s004.pdf]

Permission information of Fig 1

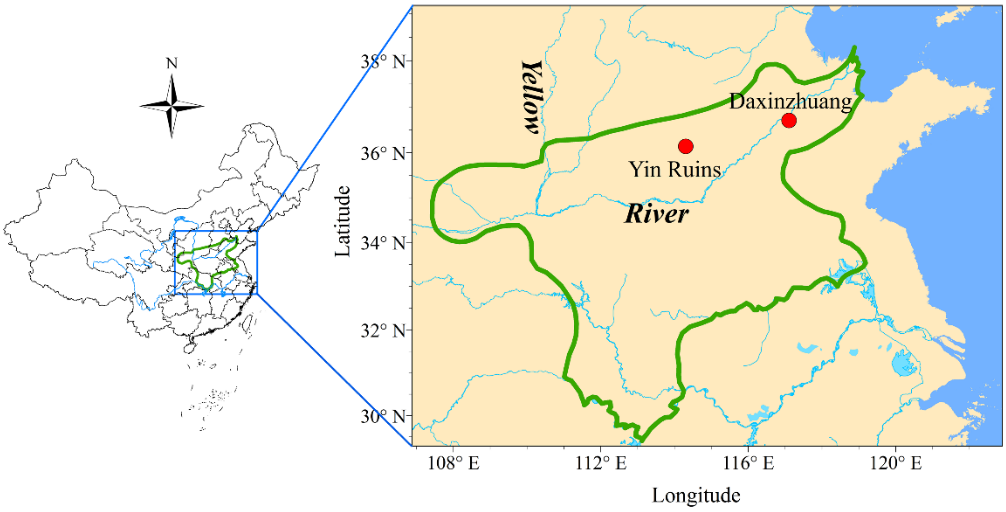

Fig 1. Map of the Shang Dynasty and location of Daxinzhuang.

The map on right was created at 24 July 2009 and the permission attribute to Lamassu Design. This file is licensed under the Creative Commons Attribution-Share Alike 3.0 Unported license (CC BY-SA 3.0). We reedited it from the original file.

Site access permissions (Original)

中华人民共和国
考古发掘证照

考执字（2011）第15号

发掘单位：山东大学
山东省文物考古研究所

发掘内容：山东省济南市历城区王舍人镇大辛庄墓地

发掘面积：1000平方米

发掘时间：2010年9月至2011年1月

发掘领队：方　辉

国家文物局

2011年1月24日

Site access permissions (Translation)

Archaeological Excavation Certificate of the People's Republic of China

(Translation)

Kaozhizi (2011)   Number : 15

Excavation Organisation:

Shandong University

Shandong Provincial Institute of Cultural Relics and Archaeology

Excavation Region:

Daxinzhuang Village, Wangsheren Town, Licheng District, Jinan City, Shandong Province.

Excavation Area:

1,000 square meters

Excavation Date: 2010-09 --- 2011.01

Excavation Director： Hui Fang

STATE ADMINISTRATION OF CULTURAL HERITAGE

2011-01-24   (Sealed)
